# Uncovering Immune Cell–Specific lncRNAs as Anti-PD-1 Response-Associated Markers in Breast Cancer

**DOI:** 10.3390/ijms27198606

**Published:** 2026-09-26

**Authors:** Clayton Voidelo, Thalita Maria do Nascimento, Regiane Stafim da Cunha, Jaqueline Carvalho de Oliveira, Carolina Mathias

**Affiliations:** 1 Post-Graduation Program in Genetics, Department of Genetics, Federal University of Paraná, Curitiba 81530-000, Brazil; 2 Department of Informatics, Federal University of Paraná, Curitiba 81530-980, Brazil; thalitanascimento@ufpr.br

**Keywords:** breast cancer, single-cell RNA-seq, lncRNA, pembrolizumab, immunotherapy

## Abstract

Long non-coding RNAs (lncRNAs) are critical regulators of the tumor microenvironment (TME); however, their cell-specific roles in predicting immunotherapy response in breast cancer (BC) remain poorly understood. This study aimed to identify immune cell-specific lncRNA signatures associated with potential pembrolizumab responsiveness by analyzing single-cell RNA sequencing data from 27 BC patients, including luminal and triple-negative (TNBC) subtypes. Differential expression analysis was performed across myeloid, lymphocyte, and dendritic cells, comparing groups based on lymphocyte expansion as a potential responsiveness criterion, classified as lymphocyte clonal expansion (E) and those without or with limited clonal expansion (NE, non-expansion). In luminal patients, potential responsiveness was primarily linked to macrophage-driven lncRNA activity. MUC20-OT1 was upregulated in responders, while LINC01094, a transcript associated with pro-tumoral M2 polarization and immune escape, was elevated in macrophages from non-responders. Conversely, TNBC responders exhibited a lymphocyte-dominant signature, characterized by the upregulation of LINC01871, LINC01943, and MIR4435-2HG. Notably, NEAT1 was uniquely identified as a significantly upregulated marker in dendritic cells of TNBC responders. While NEAT1 is known to modulate T cell abundance and thwart immune evasion in other contexts, its role in BC-associated dendritic cells remains a novel and promising area for investigation. Collectively, these findings demonstrate that lncRNA signatures in the TME are pivotal for understanding pembrolizumab sensitivity. The shift from macrophage-centered lncRNA activity in luminal subtypes to a lymphocyte- and dendritic cell-dominant profile in TNBC underscores the necessity of subtype-specific approaches. These identified lncRNAs emerge as promising candidate biomarkers associated with T cell expansion and potential response to pembrolizumab treatment to refine patient stratification and improve immunotherapeutic outcomes.

## 1. Introduction

Long non-coding RNAs (lncRNAs) are a diverse class of RNA molecules longer than 500 nucleotides, with most lacking protein-coding potential but playing critical regulatory roles in gene expression [1]. In cancer, dysregulated lncRNA expression has been associated with tumor initiation, metastasis [2], and therapeutic response [3], highlighting their potential as diagnostic biomarkers, prognostic indicators, and therapeutic targets. In particular, recent studies have demonstrated that lncRNAs expressed in specific immune cell populations can modulate immune signaling pathways and shape the tumor microenvironment, suggesting an important role in determining the efficacy of immunotherapies such as immune checkpoint inhibitors (ICIs) [4,5].

LncRNA’s roles in the tumor microenvironment (TME) are very plastic. The TME is a complex and dynamic system composed of tumor cells, extracellular matrix, stromal components, and a variety of cytokines, collectively creating conditions that support tumor growth [6]. The differential expression of lncRNAs in cells that form the TME in many cancer types has already been evaluated [7]. This variation in expression within the cells of the TME has garnered significant attention due to the advent of immunotherapies for treating various types of cancer. Identifying variations in the expression of these molecules in these cells can contribute significantly to elucidating potential markers of treatment response.

Considering the cancer types that have been benefiting from treatment with immunotherapy focused on ICIs, breast cancer (BC) stands out as a highly heterogeneous disease with significant cellular diversity in its TME composition. BC represents the most frequently diagnosed malignancy among women worldwide and the leading cause of cancer death in this population [8]. The use of ICIs in BC treatment is approved only for the triple-negative (TNBC) subtype, which is considered a “hot” tumor due to its immunogenicity [9,10]. Although the luminal subtypes are mainly considered as “cold” tumors, recent evidence demonstrates that a subset of luminal patients may present responsiveness markers to immunotherapy, suggesting that the TME of these tumors comprises complex cellular communication networks that are not yet fully elucidated [9].

Taking together lncRNA’s high tissue specificity and their documented role in regulating various oncogenic processes [11], and the fact that there is a gap in understanding the molecular mechanisms governing the response to ICIs specifically in the luminal subtypes, this study aimed to identify lncRNA signatures in TME resident cells using data from TNBC and luminal patients with differential response to ICI immunotherapy.

## 2. Results

### 2.1. lncRNAs Differentially Expressed in Myeloid Cells

We first performed cell-level differential expression analysis to identify candidate lncRNAs between patients with lymphocyte clonal expansion (E) and those without or with limited clonal expansion (NE, non-expansion) in the context of pembrolizumab treatment. These candidates were subsequently assessed at the donor level to evaluate their reproducibility across donors, applying a 10% threshold of cell fraction expression, using donor penetrance (≥70% of phenotype-group donors, or N − 1 for n ≤ 3) and Cohen’s d as criteria for supporting biological evidence, as described in Material and Methods Section 4.2.

In luminal patients (ER+ group), we identified five upregulated lncRNAs (INHBA-AS1, LINC00937, LINC01679, MUC20-OT1, and USP30-AS1) and two downregulated lncRNAs (GAPLINC and LINC01094) in myeloid cell populations when comparing those potentially responsive (presenting lymphocyte expansion, E) to pembrolizumab treatment with patients with limited benefits (non-expansion, NE), according to lymphocyte clonal expansion (Table 1). After identifying the differentially expressed lncRNAs among the analyzed groups in this specific cell lineage, we proceeded to evaluate the expression levels of these molecules in the myeloid cell (monocytes and macrophages) samples from both conditions. This information is crucial since, to be considered viable biomarkers, the molecules must exhibit a satisfactory detection level. As shown in Supplementary Figure S1A–E, the lncRNAs INHBA-AS1, LINC00937, LINC01679, GAPLINC, and USP30-AS1 displayed low detection levels, which limits their applicability as biomarkers. The lncRNAs LINC01094 and MUC20-OT1 showed interesting detection levels and were differentially expressed among the groups (Figure 1A,B). In Figure 1C,D, it is observed that LINC01094 expression is primarily concentrated within the CX3CR1, CCL2, CCR2, and MMP9 myeloid cell subpopulations. Regarding this, it is known that the CCL2/CCR2 axis is related to the monocyte’s recruitment and increased macrophage infiltration; MMP9 with tumor invasion; and CX3CR1 with an immunosuppressive phenotype [12,13,14,15]. Besides being detected in the CCR2 and CCL2 populations, MUC20-OT1 also shows detectable expression in macrophages of the LYVE1 subpopulation (Figure 1C–E). LYVE1-expressing macrophages are protumorigenic, with LYVE1 mediating hyaluronan degradation and remodeling the extracellular matrix [16].

For TNBC patients, we only identified MMP2-AS1 as differentially expressed in E group macrophages. In Figure 2A, a detectable increase in lncRNA expression is observed in the lymphocyte expansion patient group, which may suggest its potential use as a biomarker. Upon analyzing MMP2-AS1 expression across myeloid cell subpopulations, it is noted that this lncRNA is expressed in the CX3CR1, CCL18, and CCL2 subpopulations (Figure 2B,C). CCL18-derived macrophages contribute to tumor progression, including by inducing angiogenesis [17,18,19].

### 2.2. Lymphocytes Exhibited a Greater Fraction of Differentially Expressed lncRNAs

Lymphocytes are a diverse immune cell group, and, in our analysis, many lncRNAs were identified as differentially expressed in these cells in both BC analyzed subtypes. In the luminal group, MUC20-OT1 was found to be upregulated in patients with lymphocyte expansion (Table 2); however, its expression level is low considering its application as a biomarker (Supplementary Figure S2A). The lncRNAs LINC00685 and MIR3142HG (Supplementary Figure S2B,C) are downregulated in the non-expansion group, although their expression levels are low, reducing their applicability as response biomarkers.

For the TNBC subtype, seven lncRNAs were identified as upregulated in the responsive group (Table 2). Among them, three lncRNAs could be further explored considering their application as response biomarkers—LINC01871, LINC01943, and MIR4435-2HG (Figure 3A–C). Looking at the lymphocytes that showed higher expression of these lncRNAs, we observed an immunosuppressive expression pattern. LINC01871 was more expressed in exhausted T CD8 cells, LINC01943 in exhausted T CD4 cells and T CD4 regulatory cells, and MIR4435-2HG had a more diverse pattern, being expressed in T CD4 regulatory cells and exhausted T CD4 and T CD8 cells (Figure 3D–G).

### 2.3. Dendritic Cells and Differentially Expressed lncRNAs

Dendritic cells are specialized antigen-presenting cells (APCs) that act as the critical bridge between the innate and adaptive immune systems. These cells have a great impact on TME regulation that might impact ICI response. LncRNAs differential expression in dendritic cells was found only in TNBC subtype (Table 3), being NEAT1 and USP30-AS1 upregulated in patients presenting lymphocyte expansion. Considering the expression level, NEAT1 was the one that had a detectable expression value (Figure 4A). Looking at dendritic cell subpopulations, it was possible to observe that NEAT1 was not expressed only in the quiescent_migDC fraction (Figure 4B,C), with its expression being more diffuse among the other subpopulations.

### 2.4. Comparison of lncRNA Expression Profiles in Pre- and On-Treatment Samples

We evaluated differentially expressed lncRNAs between the E and NE groups in ER+ and TNBC tumors at the pre-treatment time point with pembrolizumab (Supplementary Tables S1–S3). We also compared the pre- and on-treatment time points in patients receiving pembrolizumab. Our data demonstrated increased expression of MIR4435-2HG, LINC01943, MIAT, and LINC01480 in T-lymphocyte populations from most TNBC patients with clonal expansion (E group) (Supplementary Figure S9). In contrast, most ER+ patients in the group E showed reduced LINC01094 expression in myeloid cells (Supplementary Figure S10). The other lncRNAs analyzed either did not show statistically significant differences between the time points or did not exhibit a consistent pattern of increased or decreased expression across patients.

## 3. Discussion

The profile of differentially expressed lncRNAs in immune cells among luminal and TNBC BC subtypes may reveal potential response biomarkers to pembrolizumab treatment. Based on previously published data [13], our objective was to identify lncRNAs that could serve as potential biomarkers of response to anti-PD-1 therapy. In the original study, the authors focused primarily on mRNAs. Given the important role of lncRNAs as regulators of gene expression, we sought to extend these findings by highlighting lncRNAs within the immune cell populations previously annotated by the authors.

In luminal patients, our findings highlight an enhanced contribution of myeloid cells, including macrophage and monocyte populations, to lncRNA differential expression in the context of pembrolizumab responsiveness related to lymphocyte clonal expansion criteria. Specifically, MUC20-OT1 expression was elevated in myeloid cells from responders, whereas LINC01094 expression was increased in those from non-responders. In contrast, TNBC patients exhibit a higher number of detected lncRNAs in relation to pembrolizumab treatment response in lymphocytes. Considering the expression level, our main findings in TNBC patients include increased expression of LINC01871, LINC01943, and MIR4435-2HG in lymphocytes, MMP2-AS1 in macrophages, and NEAT1 in dendritic cells; all were detected in the treatment-responsive group. Although differences were observed between the groups, only a few of the lncRNAs evaluated changes between the pre- and on-treatment time points. Thus, caution is warranted when interpreting these changes as being directly associated with pembrolizumab treatment.

LINC01094 has been consistently implicated in promoting tumor progression, including pathway modulation related to tumor growth, invasion, and metastasis [20,21]. In luminal non-expansion patients, we found a higher expression of LINC01094 in myeloid cells, particularly in the CX3CR1, CCL2, CCR2, and MMP9 subpopulations. In luminal potentially responsive patients with lymphocyte clonal expansion, we also observed reduced LINC01094 expression at the on-treatment with pembrolizumab time point compared with the pre-treatment time point in two of the three patients. Higher levels of LINC01094 in tumor tissue have also been associated with a worse prognosis in several types of cancer, including breast, gastric and bladder cancers [21,22,23]. Regarding the immune response in the tumor, LINC01094 expression has been associated with increased macrophage infiltration [21,23], especially those with an immunosuppressive and pro-tumoral role, such as M2 and IL-10^+^ TAM [24]. Among the myeloid cell subpopulations with higher LINC01094 expression analyzed in our study, the CCL2/CCR2 axis is related to monocyte recruitment and higher macrophage infiltration and tends to polarize towards the M2 phenotype [12,14,25]. In vitro data demonstrated that LINC01094 binds SPI1, a transcriptional factor, promoting its nuclear translocation and enhancing *CCL7* transcription, which in turn drives macrophage chemotaxis toward the TME [26]. Furthermore, macrophage subpopulations expressing MMP9 are involved in extracellular matrix remodeling, promoting tumor invasion [15]. In addition, LINC01094 regulates PD-L1/PD-L2 expression through a ceRNA axis binding miR-17-5p, preventing this miRNA from degrading the PD-L1/PD-L2 transcripts [24]. Although we found no difference in LINC01094 expression in lymphocytes between the lymphocyte expansion and non-expansion groups, LINC01094 is also associated with CD8+ T cell exhaustion [24]. Notably, the CX3CR1 macrophage subpopulation exhibits elevated LINC01094 expression which is also associated with reduced T-lymphocyte infiltration and a protumorigenic phenotype [13,27]. Taken together, these data suggest that LINC01094 plays a role modulating immune responses mediated by myeloid cells, particularly macrophages and monocytes.

Our data indicates that MUC20-OT1 levels are elevated in both myeloid cells and lymphocytes in luminal patients with lymphocyte expansion. A study conducted by Lin et al. (2024) demonstrated that MUC20-OT1 expression is upregulated in hepatocellular carcinoma compared to normal tissue, based on bulk RNA-seq analysis [28]. Notably, higher expression of MUC20-OT1 is associated with poorer relapse-free survival [28]. Despite this, this lncRNA remains poorly characterized to date, and its function and potential role in cancer and other diseases are still unclear. The macrophage subpopulation with the highest MUC20-OT1 expression is marked by LYVE1 expression, which has also been described as having a protumorogenic role [16]. LYVE1 is a glycoprotein that induces hyaluronan degradation, modulating the extracellular matrix, especially near the lymphatic vessels [16,29]. Interestingly, data from an animal model of BC have shown that blocking this population enhances CD8+ T cell recruitment and improves the response to chemotherapy [16,29].

Myeloid cells from TNBC patients with lymphocyte expansion exhibit increased MMP2-AS1 expression. Although there is no data in the literature to date indicating the potential role of this lncRNA in immune cells, some studies have reported its association with autophagy pathways [30,31,32]. Based on bulk RNA-seq data, lower levels of MMP2-AS1 were observed in lung adenocarcinoma tumors compared to normal tissue [31]. Another study demonstrated that higher levels of MMP2-AS1 in tumor tissue are associated with better overall survival in patients with non-small cell lung cancer [32]. In contrast, in vitro evidence suggests that MMP2-AS1 upregulation may also promote tumor progression. MMP2-AS1 has been found to contribute to proliferation, migration, and invasion in renal cell carcinoma cells by targeting miR-34c-5p, which elevates MMP2 expression, a protein involved in extracellular matrix degradation [33]. Interestingly, our data demonstrated that MMP2-AS1 is more expressed in myeloid cell subpopulations expressing CCL18, CCL2, and CX3CR1. In addition to the CCL2 and CX3CR1 subpopulations already discussed, CCL18 macrophages are also associated with tumor progression, metastasis, and poorer prognosis [17,18,19]. Therefore, there is still a gap in understanding the role of this lncRNA in macrophage subpopulations and, consequently, within the TME.

Lymphocytes were the cells analyzed with the most differentially expressed lncRNAs in TNBC patients following pembrolizumab treatment. Among the identified lncRNAs, LINC01871, LINC01943, and MIR4435-2HG are particularly notable for their high abundance, enhancing their potential as detectable biomarkers. All three are upregulated in the potentially responsive group with lymphocyte expansion compared to the non-expansion group. The three lncRNAs have already been described as participating in immune pathways in cancer. LINC01871 has already been evidenced in previous work by our research group that it is related to immune response activation and favorable overall survival in basal-like samples [34]. This lncRNA was also discussed by Zhou and colleagues (2024) as an element of a risk signature in immunotherapy’s effectiveness for BC [35]. LINC01943 was described as composing an immune-related lncRNA and was associated with better prognosis in TNBC patients [36]. Finally, MIR4435-2HG was also an element of an immune-related lncRNA that predicts prognosis in BC patients [37], and an in vitro assay showed that MIR4435-2HG might promote the proliferation and migration of BC cells, facilitate the polarization of M1 into M2 macrophages, enhance the migration of macrophages and increase the PD-1/PD-L1/CTLA4 expression [38]. Interestingly, both LINC01943 and MIR4435-2HG showed increased expression in lymphocytes at the on-treatment time point compared with the pre-treatment time point in most TNBC patients with lymphocyte expansion, with this pattern observed in four of the five patients.

Considering dendritic cells, only for TNBC, we found statistically differentiated lncRNAs—NEAT1 and USP30-AS1, with NEAT1 being the one that showed higher expression levels. NEAT1 is a crucial element for paraspeckles organization [39], and its role in several cancer hallmarks has also been investigated in BC [40]. The role of NEAT in immune response in BC is gaining more attention. In a study conducted by Xiao et al. (2025), NEAT1 silencing the treatment thwarts BC immune escape through the induction of active CD8+ and CD3+ T cells [41]. Additionally, it reverses the immunosuppressive landscape by polarizing TAMs from an M2 to an M1 state. Beyond inhibiting EMT, this strategy demonstrates a synergistic effect when combined with anti-PD-1 immune checkpoint blockade. NEAT1 expression was also correlated with CD8 T cell abundance in BC [3]. In dendritic cells, Zhang and colleagues (2019), using a knockdown system, verified that NEAT can induce a tolerogenic phenotype in dendritic cells [42]. NEAT1 remains unexplored regarding BC, which presents a highly interesting area for assessment.

## 4. Material and Methods

### 4.1. scRNA-Seq Dataset Acquisition

In this study, we used a publicly available single-cell RNA-seq dataset generated in the BioKey clinical study (NCT03197389), first reported by Bassez et al. (2021) [13]. The dataset comprises scRNA-seq data from BC patients (from both TNBC and luminal subtypes) treated with pembrolizumab as a monotherapy. Expression data were obtained before and after treatment, and patients were considered potentially responsive to the treatment based on lymphocyte expansion criteria. To focus on active microenvironmental changes after treatment exposure, our analyses were conducted only in on-treatment biopsies. The study evaluated 27 BC samples, with 12 being classified as TNBC and 15 as luminal patients. The dataset includes pre-existing immune cell annotations; therefore, the analyses performed in this study focused on myeloid, lymphocyte, and dendritic cell populations, the most detailed group of cells available.

### 4.2. Differential Expression Analysis and lncRNAs Annotation

The differential expression analysis was performed using the Seurat package [43] and using data from immune cells—myeloid (monocytes and macrophages), lymphocytes and dendritic cells in the on-treatment cohort. We evaluated the differential expression within each isolated cell lineage independently among each subtype (TNBC and luminal) considering expansion (E), the potentially responsive patients to pembrolizumab, and non-expansion (NE), patients with limited benefits to the treatment, based on the presence or limited extent of clonal lymphocyte expansion, respectively. For TNBC, 7 patients were classified as non-expansion and 5 as presenting lymphocyte expansion, and for luminal patients 12 were considered as non-expansion and 3 as lymphocyte expansion. Single-cell comparisons were executed using the two-sided Wilcoxon rank-sum test via Seurat’s *FindMarkers()* function, and we adopted statistical significance as a Bonferroni-adjusted *p*-value < 0.05 and |avg_log2FC| > 1. Regarding sequencing depth, prior to the differential analysis in Seurat, we followed the vignette pipeline and normalized the global differences in sequence depth and library size using the *LogNormalize* function algorithm (which works with a standard scale factor of 10,000); it scales transcript counts relative to total cellular mRNA output (https://satijalab.org/seurat/articles/pbmc3k_tutorial (accessed on 22 September 2026)).

LncRNAs presented among differentially expressed genes were annotated and filtered using biomaRt package (version 3.23) [44] by selecting transcripts with the “lncRNA” gene biotype, based on updated transcript guidelines [1]. To ensure the clinical potential of the identified lncRNAs as biomarkers, we applied a second filtering based on lncRNAs’ cell expression fraction in both analyzed groups. We used, as a threshold, at least 10% of expression (top 10% cell expression fraction), selecting those with consistent distribution in violin plots, avoiding candidates with high dropout rates or expression restricted to a negligible number of cells. To prevent cell-level pseudoreplication, candidates were restricted to lncRNAs with robust donor penetrance (≥70% of phenotype group donors, or N − 1 for n ≤ 3) and validated through post hoc sensitivity testing of patient-averaged expression values coupled with Cohen’s d effect size estimation (Supplementary Figures S3–S8).

### 4.3. Paired Pre-Treatment vs. On-Treatment Patient Evaluation

In order to evaluate if the lncRNA candidates identified in the on-treatment cohort during the exploratory discovery step displayed alterations in expression levels upon pembrolizumab exposure rather than only being associated with lymphocyte expansion criteria, we implemented a donor-level paired analysis, matching pre- and on-treatment biopsies from the 27 selected breast cancer patients. For each lncRNA candidate, we extracted log-normalized expression values per patient at each time point within each specific cell lineage (myeloid, lymphocyte, and dendritic cells). The paired changes in expression between pre- and on-treatment were assessed using a two-sided Wilcoxon rank-sum test (Supplementary Figures S9 and S10).

## 5. Conclusions

Our findings demonstrate that lncRNA signatures in the immune cells from the TME may be pivotal to understanding pembrolizumab resistance and sensitivity. We highlight a shift from macrophage-centered lncRNA activity in luminal subtypes to a lymphocyte- and dendritic cell-dominant profile in TNBC responders. Key lncRNAs, such as LINC01094 in non-expansion luminal cases and NEAT1 or LINC01871 in TNBC cases with lymphocyte expansion, emerge as promising candidate biomarkers for a potential pembrolizumab response. We also highlight the limitations of this study, including the small number of patients analyzed and the imbalance between group identities, especially in ER+ samples (which reflects the intrinsic biology of luminal breast tumors as immunologically “cold” microenvironments with characteristically low rates of response and T cell clonal expansion under immunotherapy). Further studies with larger cohorts are warranted to confirm and validate our findings. Importantly, because our profiling focused on on-treatment biopsies, the identified candidates should be interpreted as response-associated signatures rather than predictive biomarkers. Future longitudinal studies tracking paired pre- and post-treatment cohorts, correlating lncRNA expression with more classical clinical outcome data beyond T lymphocyte expansion, will be necessary to determine whether basal expression of these transcripts holds true predictive utility prior to immunotherapy initiation. These lncRNAs should be explored as signatures in functional assays in order to elucidate their molecular mechanisms associated with those immune cell subpopulations in the anti-PD-1 response. By elucidating these subtype-specific immune modulations, this study provides a foundation for the understanding of personalized immunotherapeutic strategies aimed at overcoming immunosuppression and improving survival outcomes in breast cancer.

## Figures and Tables

**Figure 1:**
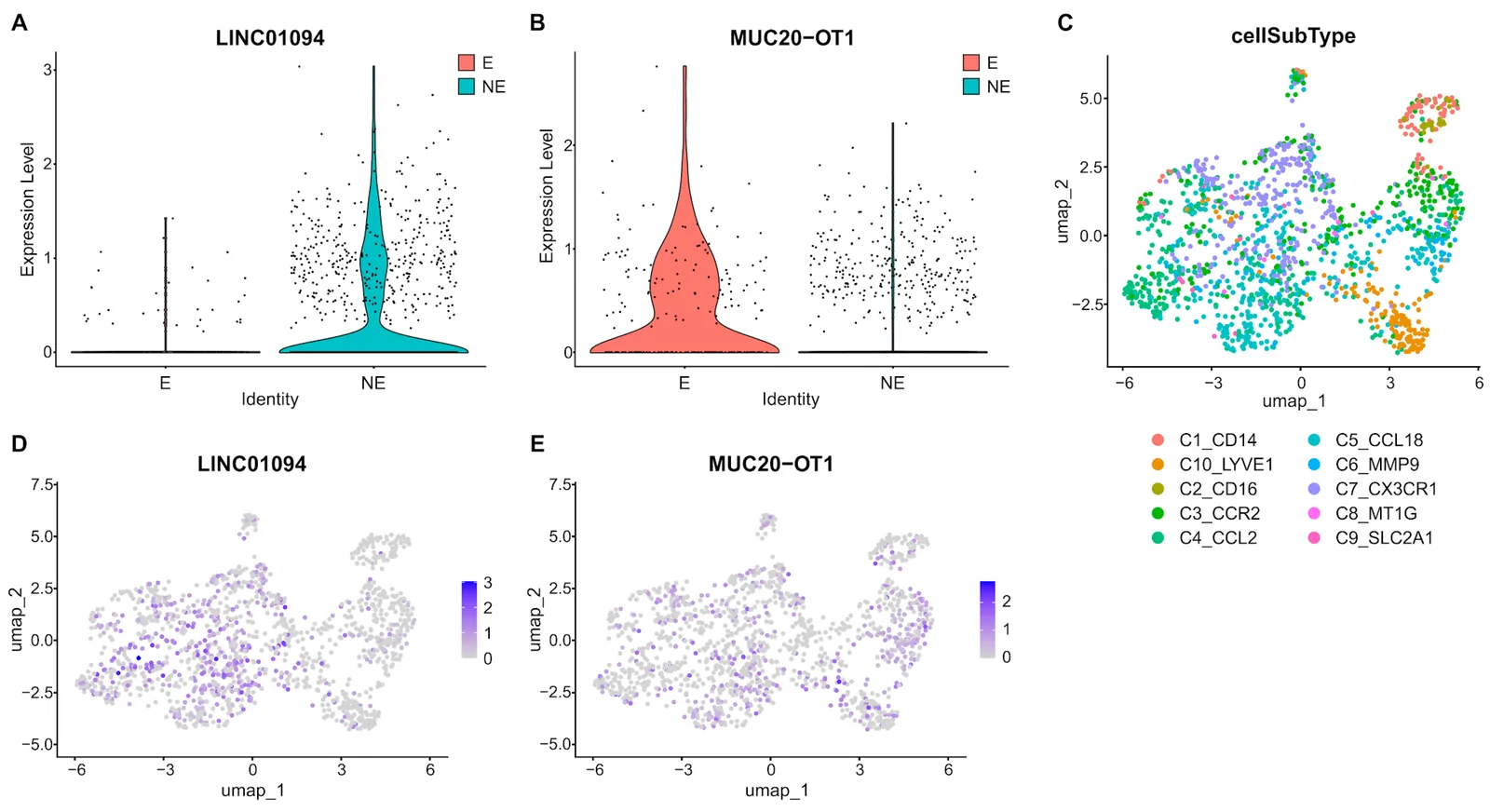
LINC01094 and MUC20-OT1 expression in the myeloid cell population of patients with luminal breast cancer on-pembrolizumab treatment. Violin plots showing the expression of (**A**) LINC01094 and (**B**) MUC20-OT1 in E (lymphocyte expansion) and NE (non-expansion) groups. (**C**) UMAP of the overall myeloid cell population. UMAPs highlighting myeloid cell subpopulations expressing (**D**) LINC01094 and (**E**) MUC20-OT1 in the E group compared to the NE group. E, potentially responsive to pembrolizumab treatment due to lymphocyte expansion; NE, potentially non-responsive to pembrolizumab treatment due to limited lymphocyte expansion; C1–C10, distinct macrophage subpopulations.

**Figure 2:**
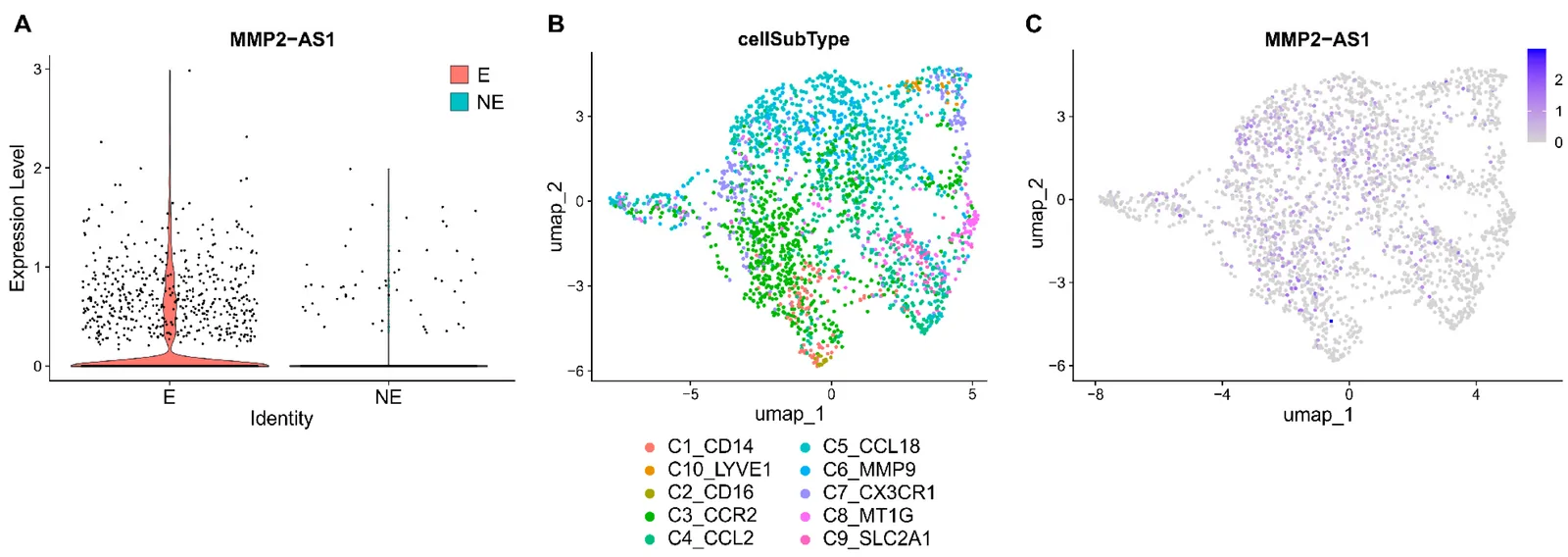
MMP2-AS1 expression in the myeloid cell population of patients with triple-negative breast cancer subtype on-pembrolizumab treatment. Violin plot showing the expression of (**A**) MMP2-AS1 in E (lymphocyte expansion) and NE (non-expansion) groups. UMAPs of (**B**) the overall myeloid cell population and (**C**) the subpopulations expressing MMP2-AS1 in the E group compared with the NE group. E, potentially responsive to pembrolizumab treatment due to lymphocyte expansion; NE, potentially non-responsive to pembrolizumab treatment due to limited lymphocyte expansion; C1–C10, distinct macrophage subpopulations.

**Figure 3:**
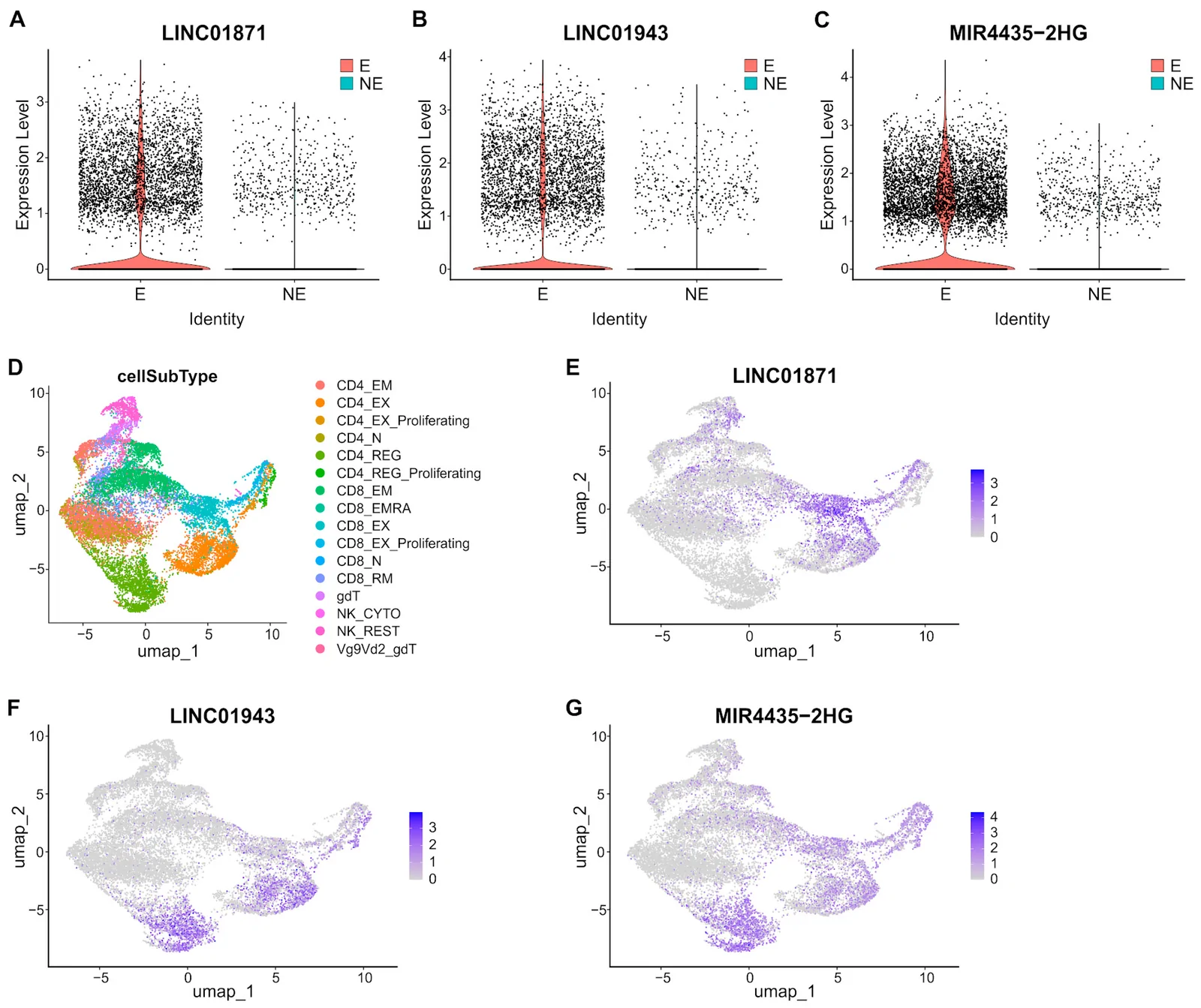
LINC01871, LINC01943 and MIR4435-2HG expression in the lymphocyte population of patients with triple-negative breast cancer subtype on-pembrolizumab treatment. Violin plot showing the expression of (**A**) LINC01871, (**B**) LINC01943, and (**C**) MIR4435-2HG in E (lymphocyte expansion) and NE (non-expansion) groups. (**D**) UMAP of the overall lymphocyte population. UMAPs highlight the subpopulations expressing (**E**) LINC01871, (**F**) LINC01943, and (**G**) MIR4435-2HG in the E group compared with the NE group. E, potentially responsive to pembrolizumab treatment due to lymphocyte expansion; NE, potentially non-responsive to pembrolizumab treatment due to limited lymphocyte expansion.

**Figure 4:**
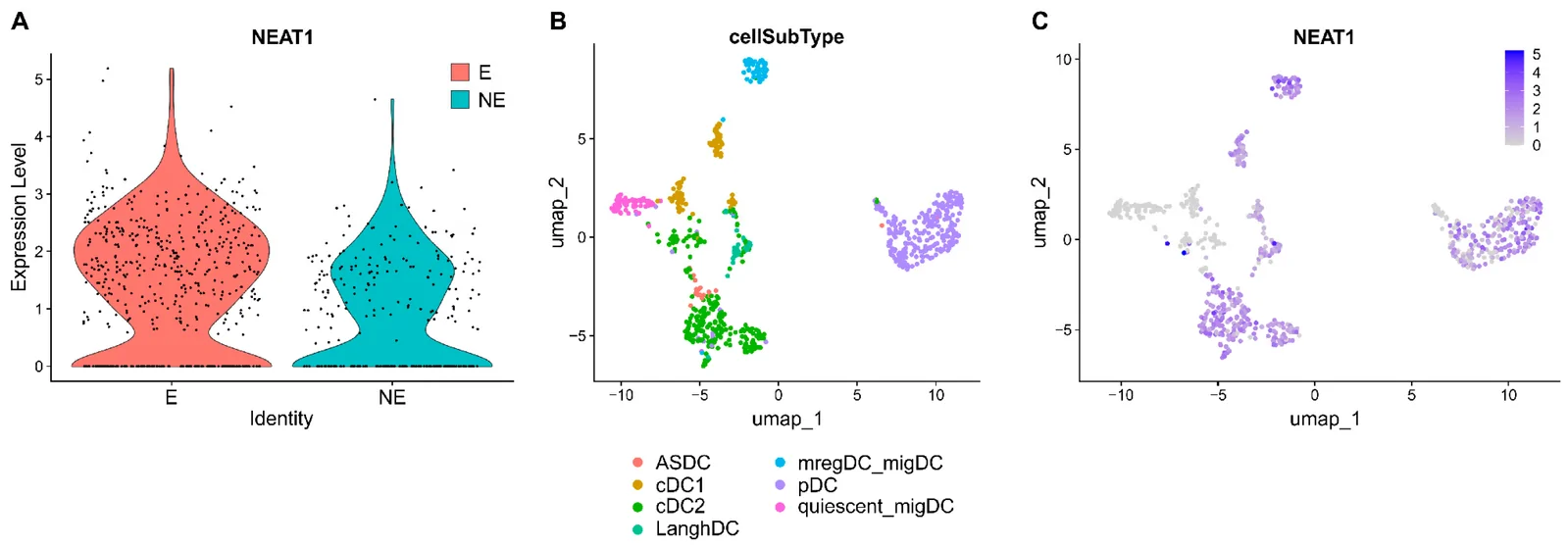
NEAT1 expression in the dendritic cells of patients with triple-negative breast cancer subtype on-pembrolizumab treatment. Violin plot showing the expression of (**A**) NEAT1 in E (lymphocyte expansion) and NE (non-expansion) groups. UMAPs of (**B**) the overall dendritic cell population and (**C**) the subpopulations expressing NEAT1 in the E group compared with the NE group. E, potentially responsive to pembrolizumab treatment due to lymphocyte expansion; NE, potentially non-responsive to pembrolizumab treatment due to limited lymphocyte expansion.

**Table 1:** LncRNAs expressed by myeloid cell populations identified in scRNA-seq data from ER+ and TNBC patients treated with pembrolizumab in on-treatment time point.

BRCA Subtype	lncRNAs	Average log2 Fold Changes	*p*-Value Adj	Cell Expression Fraction (%)	
				E	NE
ER+	INHBA-AS1	3.7879	6.82 × 10^−4^	4.80	0.50
	LINC01679	2.8921	2.17 × 10^−8^	7.50	0.70
	USP30-AS1	1.6782	1.01 × 10^−5^	16.30	4.80
	LINC00937	1.6586	3.78 × 10^−5^	16.30	5.10
	MUC20-OT1	1.0028	7.44 × 10^−7^	48.00	25.00
	GAPLINC	−5.2646	3.06 × 10^−5^	0.40	14.90
	LINC01094	−2.5373	1.55 × 10^−6^	14.10	33.10
TNBC	MMP2-AS1	1.2539	3.50 × 10^−14^	28.00	9.40

Note: The total number of cells evaluated was 227 in the ER+ E group, 1178 in the ER+ NE group, 1805 in the TNBC E group, and 573 in the TNBC NE group. E, potentially responsive to pembrolizumab treatment due to lymphocyte expansion; NE, potentially non-responsive to pembrolizumab treatment due to limited lymphocyte expansion.

**Table 2:** LncRNAs expressed by lymphocyte populations identified in scRNA-seq data from ER+ and TNBC patients treated with pembrolizumab in on-treatment time point.

BRCA Subtype	lncRNAs	Average log2 Fold Changes	*p*-Value Adj	Cell Expression Fraction (%)	
				E	NE
ER+	MUC20-OT1	1.0506	1.72 × 10^−32^	20.30	10.80
	MIR3142HG	−1.3199	8.09 × 10^−11^	2.40	5.50
	LINC00685	−2.1730	4.43 × 10^−5^	0.30	1.30
TNBC	MIR4435-2HG	1.6380	2.99 × 10^−150^	38.40	15.30
	LINC01943	1.6405	3.30 × 10^−85^	25.70	10.40
	LINC01871	1.1934	9.79 × 10^−55^	26.70	14.00
	MIAT	1.2904	6.17 × 10^−53^	19.10	8.00
	LINC02446	1.2687	1.17 × 10^−38^	18.20	9.00
	MIR155HG	1.0929	7.12 × 10^−27^	18.90	10.80
	LINC01480	1.1924	1.96 × 10^−21^	7.40	2.60
	LINC00402	−1.3087	7.57 × 10^−20^	2.80	6.30

Note: The total number of cells evaluated was 4931 in the ER+ E group, 4451 in the ER+ NE group, 12,041 in the TNBC E group, and 3789 in the TNBC NE group. E, potentially responsive to pembrolizumab treatment due to lymphocyte expansion; NE, potentially non-responsive to pembrolizumab treatment due to limited lymphocyte expansion.

**Table 3:** LncRNAs expressed by dendritic cell populations identified in scRNA-seq data from ER+ and TNBC patients treated with pembrolizumab in on-treatment time point.

BRCA Subtype	lncRNAs	Average log2 Fold Changes	*p*-Value Adj	Cell Expression Fraction (%)	
				E	NE
ER+	NA	NA	NA	NA	NA
TNBC	NEAT1	1.0071	6.66 × 10^−6^	74.50	60.00
	USP30-AS1	2.0459	7.30 × 10^−3^	18.50	5.10

Note: The total number of cells evaluated was 178 in the ER+ E group, 263 in the ER+ NE group, 530 in the TNBC E group, and 255 in the TNBC NE group. E, potentially responsive to pembrolizumab treatment due to lymphocyte expansion; NE, potentially non-responsive to pembrolizumab treatment due to limited lymphocyte expansion; NA: no lncRNAs met our identification thresholds.

## Data Availability

The original contributions presented in this study are included in the article/Supplementary Materials. Further inquiries can be directed to the corresponding author.

## Supplementary Materials

The following supporting information can be downloaded at: https://www.mdpi.com/article/10.3390/ijms27198606/s1.
