# Supplementary material for: Uncovering Immune Cell–Specific lncRNAs as Anti-PD-1 Response-Associated Markers in Breast Cancer
Source: Int J Mol Sci. 2026 Sep 26;27(19):8606. doi: 10.3390/ijms27198606 (PMC13664770; doi:10.3390/ijms27198606)
Supplement: Supplementary file 1 [file ijms-27-08606-s001.zip › ijms-4539025-supplementary.pdf]

Supplementary Material

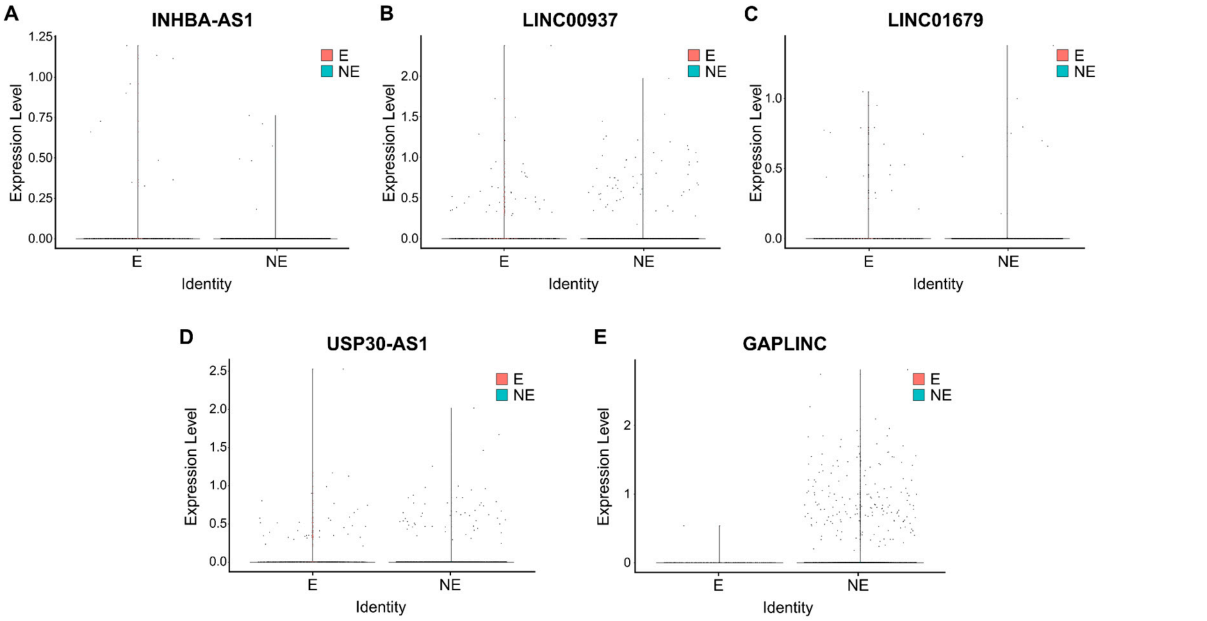

Supplementary Figure S1. INHBA-AS1, LINC00937, LINC01679, USP30-AS1 and GAPLINC expression in the macrophage population of patients with luminal breast cancer subtype receiving pembrolizumab treatment. Violin plots showing the expression of (A) INHBA-AS1, (B) LINC00937, (C) LINC01679, (D) USP30-AS1 and (E) GAPLINC in the lymphocyte expansion (E) and limited lymphocyte expansion (NE) groups.

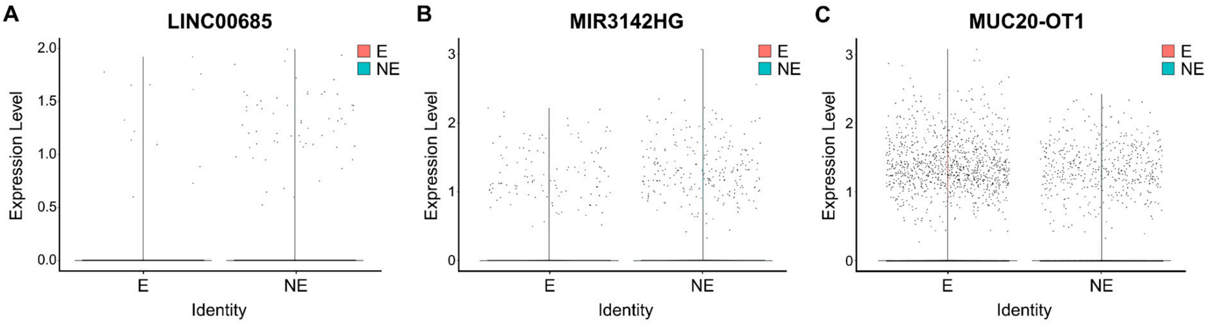

Supplementary Figure S2. LINC00685, MIR3142HG and MUC20-OT1 expression in the lymphocyte population of patients with luminal breast cancer subtype receiving pembrolizumab treatment. Violin plots showing the expression of (A) LINC00685, (B) MIR3142HG, and (C) MUC20-OT1 in E and NE groups. E, potentially responsive to Pembrolizumab treatment due to lymphocyte expansion; NE, potentially non-responsive to Pembrolizumab treatment due to limited lymphocyte expansion.

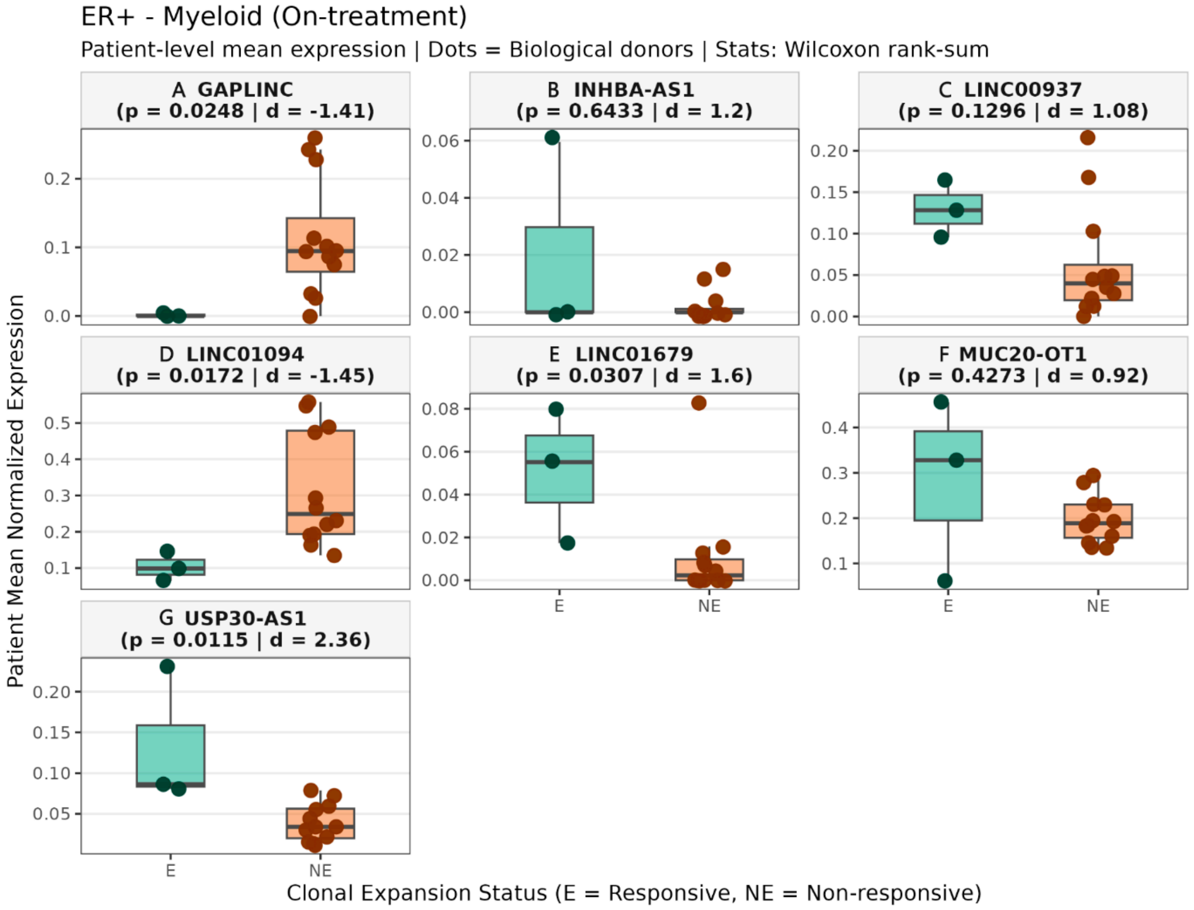

Supplementary Figure S3. Donor-level mean expression of candidate lncRNAs in the myeloid cell population of patients with ER+ breast cancer receiving on-treatment pembrolizumab. Hybrid boxplots overlaid with individual donor jitter points showing the patient-averaged normalized expression of **(A)** GAPLINC, **(B)** INHBA-AS1, **(C)** LINC00937, **(D)** LINC01094, **(E)** LINC01679, **(F)** MUC20-OT1, and **(G)** USP30-AS1 in the lymphocyte expansion (E) and limited lymphocyte expansion (NE) groups. Each point represents the mean expression of an individual biological donor. Statistical significance between groups was evaluated using a two-sided Wilcoxon rank-sum test at the patient level (p), and biological effect magnitude was quantified using Cohen's d effect size (d). E, potentially responsive to Pembrolizumab treatment due to lymphocyte expansion; NE, potentially non-responsive to Pembrolizumab treatment due to limited lymphocyte expansion.

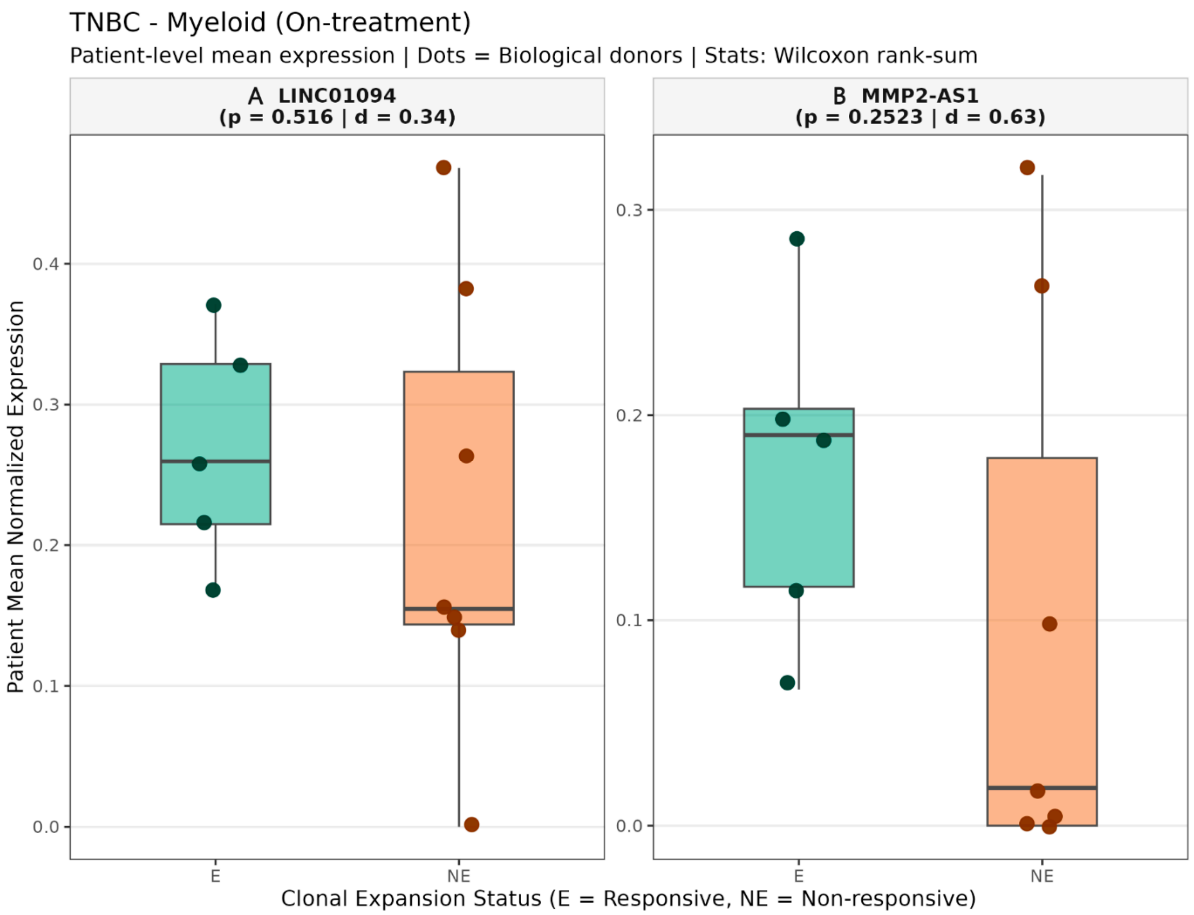

Supplementary Figure S4. Donor-level mean expression of candidate lncRNAs in the myeloid cell population of patients with triple-negative breast cancer (TNBC) receiving on-treatment pembrolizumab. Hybrid boxplots overlaid with individual donor jitter points showing the patient-averaged normalized expression of **(A)** LINC01094 and **(B)** MMP2-AS1 in the lymphocyte expansion (E) and limited lymphocyte expansion (NE) groups. Each point represents the mean expression of an individual biological donor. Statistical significance between groups was evaluated using a two-sided Wilcoxon rank-sum test at the patient level (p), and biological effect magnitude was quantified using Cohen's d effect size (d). E, potentially responsive to Pembrolizumab treatment due to lymphocyte expansion; NE, potentially non-responsive to Pembrolizumab treatment due to limited lymphocyte expansion.

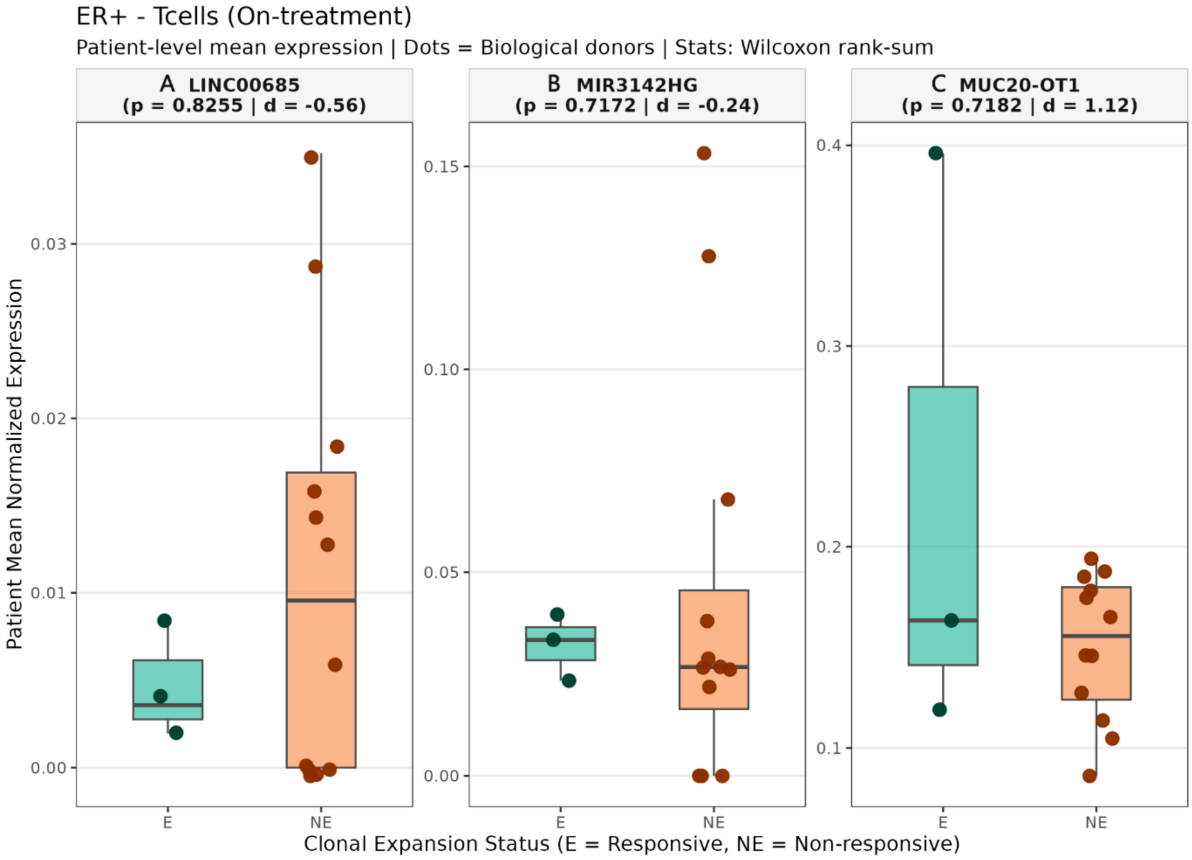

Supplementary Figure S5. Donor-level mean expression of candidate lncRNAs in the T-cell population of patients with ER+ breast cancer receiving on-treatment pembrolizumab. Hybrid boxplots overlaid with individual donor jitter points showing the patient-averaged normalized expression of **(A)** LINC00685, **(B)** MIR3142HG, and **(C)** MUC20-OT1 in the lymphocyte expansion (E) and limited lymphocyte expansion (NE) groups. Each point represents the mean expression of an individual biological donor. Statistical significance between groups was evaluated using a two-sided Wilcoxon rank-sum test at the patient level (p), and biological effect magnitude was quantified using Cohen's d effect size (d). E, potentially responsive to Pembrolizumab treatment due to lymphocyte expansion; NE, potentially non-responsive to Pembrolizumab treatment due to limited lymphocyte expansion.

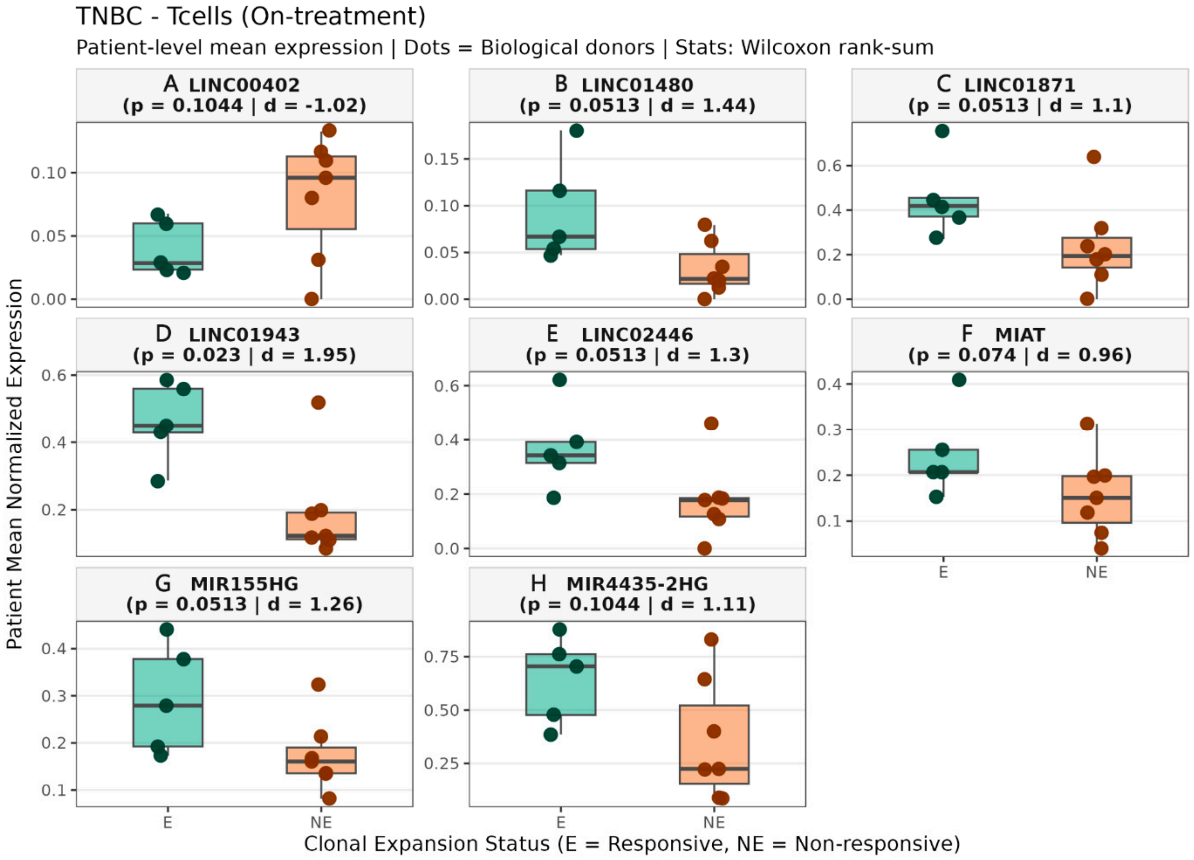

Supplementary Figure S6. Donor-level mean expression of candidate lncRNAs in the T-cell population of patients with triple-negative breast cancer (TNBC) receiving on-treatment pembrolizumab. Hybrid boxplots overlaid with individual donor jitter points showing the patient-averaged normalized expression of **(A)** LINC00402, **(B)** LINC01480, **(C)** LINC01871, **(D)** LINC01943, **(E)** LINC02446, **(F)** MIAT, **(G)** MIR4435-2HG, and **(H)** MIR4435-2HGx in the lymphocyte expansion (E) and limited lymphocyte expansion (NE) groups. Each point represents the mean expression of an individual biological donor. Statistical significance between groups was evaluated using a two-sided Wilcoxon rank-sum test at the patient level (p), and biological effect magnitude was quantified using Cohen's d effect size (d). E, potentially responsive to Pembrolizumab treatment due to lymphocyte expansion; NE, potentially non-responsive to Pembrolizumab treatment due to limited lymphocyte expansion.

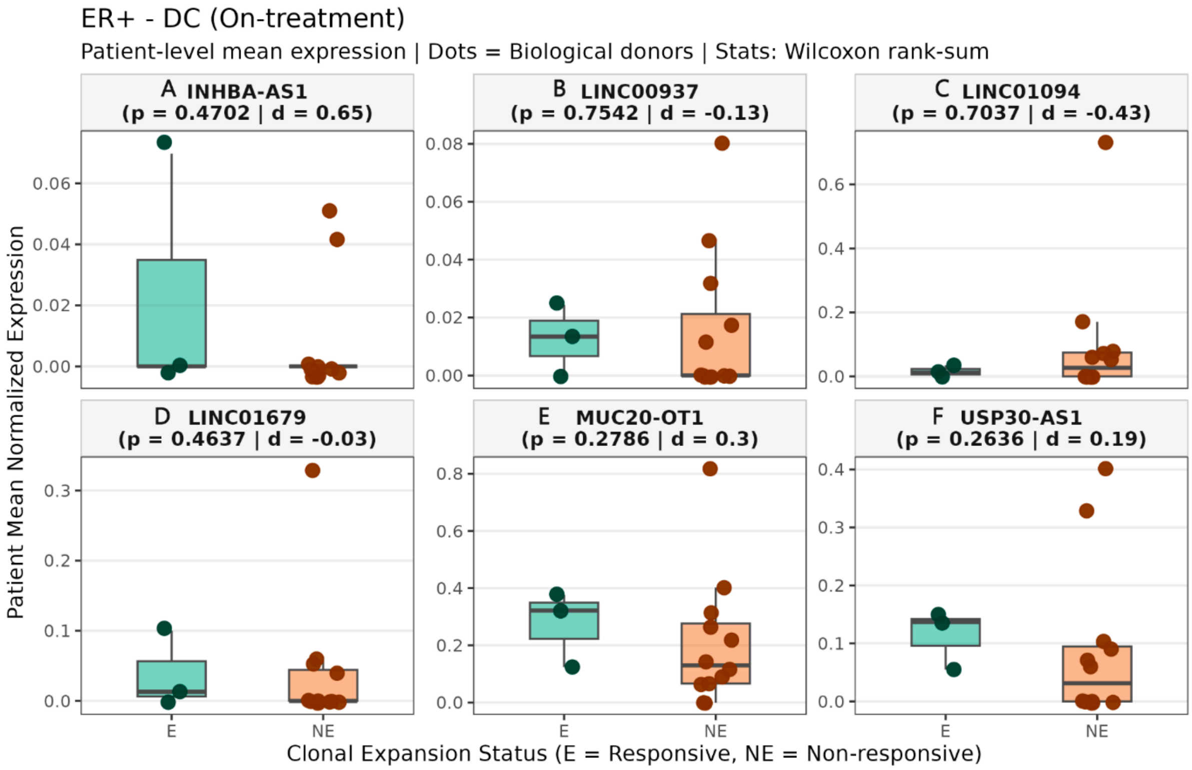

Supplementary Figure S7. Donor-level mean expression of candidate lncRNAs in the dendritic cell population of patients with ER+ breast cancer receiving on-treatment pembrolizumab. Hybrid boxplots overlaid with individual donor jitter points showing the patient-averaged normalized expression of **(A)** INHBA-AS1, **(B)** LINC00937, **(C)** LINC01679, **(D)** MUC20-OT1, **(E)** USP30-AS1, **(F)** GAPLINC, and **(G)** LINC01094 in the lymphocyte expansion (E) and limited lymphocyte expansion (NE) groups. Each point represents the mean expression of an individual biological donor. Statistical significance between groups was evaluated using a two-sided Wilcoxon rank-sum test at the patient level (p), and biological effect magnitude was quantified using Cohen's d effect size (d). E, potentially responsive to Pembrolizumab treatment due to lymphocyte expansion; NE, potentially non-responsive to Pembrolizumab treatment due to limited lymphocyte expansion.

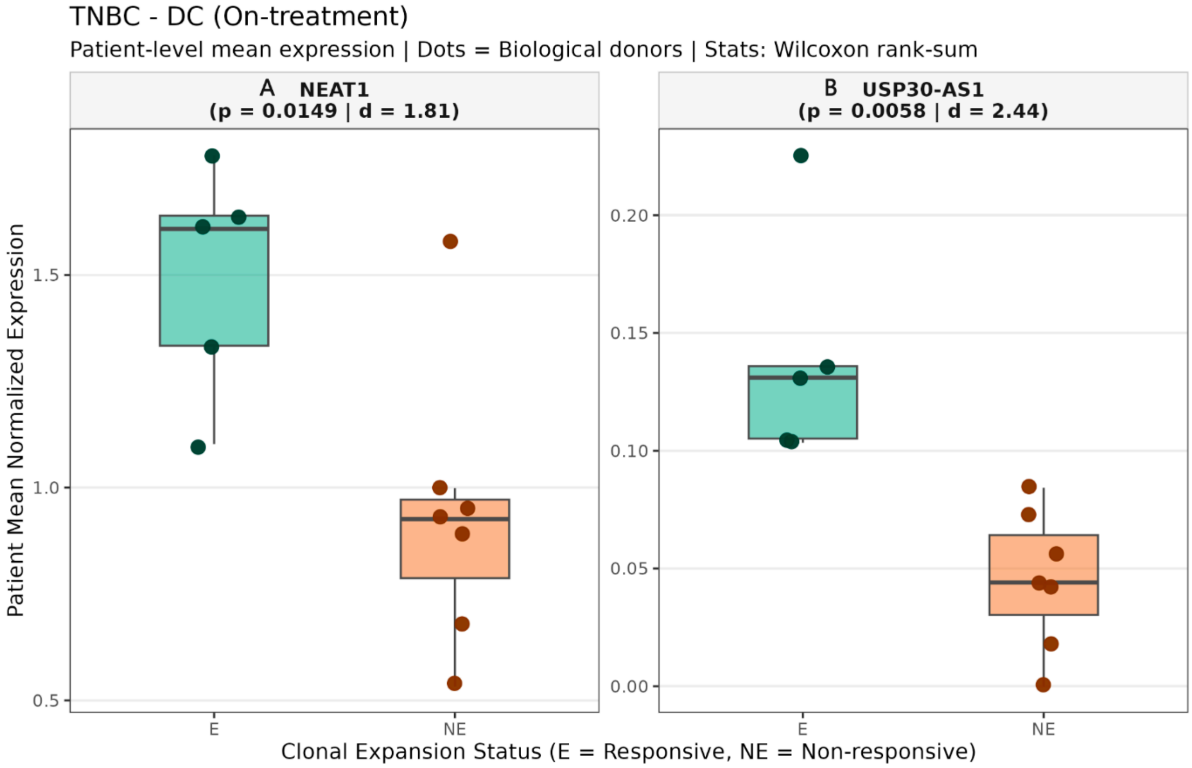

Supplementary Figure S8. Donor-level mean expression of candidate lncRNAs in the dendritic cell population of patients with triple-negative breast cancer (TNBC) receiving on-treatment pembrolizumab. Hybrid boxplots overlaid with individual donor jitter points showing the patient-averaged normalized expression of **(A)** NEAT1 and **(B)** USP30-AS1 in the lymphocyte expansion (E) and limited lymphocyte expansion (NE) groups. Each point represents the mean expression of an individual biological donor. Statistical significance between groups was evaluated using a two-sided Wilcoxon rank-sum test at the patient level (p), and biological effect magnitude was quantified using Cohen's d effect size (d). E, potentially responsive to Pembrolizumab treatment due to lymphocyte expansion; NE, potentially non-responsive to Pembrolizumab treatment due to limited lymphocyte expansion.

Supplementary Table S1. LncRNAs expressed by myeloid populations identified in scRNA-seq data from ER+ and TNBC patients treated with pembrolizumab in pre-treatment time point.

BRCA subtype	lncRNAs	Average log2 fold changes	p-value adj	Cell expression fraction (%)	
				E	NE
ER+	*NA*	*NA*	*NA*	*NA*	*NA*
TNBC	LINC01857	1.25619	2.84E-02	13.08	5.70
	LINC02345	1.42056	2.13E-02	11.70	4.10
	MMP2-AS1	1.17075	2.28E-03	20.02	9.80
	ATP2B1-AS1	-1,04020	6.95E-14	34.08	5.40
	LINC00877	-1,43659	2.88E-02	2.70	7.70
	LINC01480	-2,16987	4.60E-03	2.80	8.30

Note: The total number of cells evaluated was 137 in the ER+ E group and 735 in the ER+ NE group; and 1,260 in the TNBC E group and 509 in the TNBC NE group. E, potentially responsive to Pembrolizumab treatment due to lymphocyte expansion; NE, potentially non-responsive to

Pembrolizumab treatment due to limited lymphocyte expansion. *NA*: No lncRNAs met our identification thresholds.

Supplementary Table S2. LncRNAs expressed by lymphocyte populations identified in scRNA-seq data from ER+ and TNBC patients treated with pembrolizumab in pre-treatment time point.

BRCA subtype	lncRNAs	Average log2 fold changes	p-value adj	Cell expression fraction (%)	
				E	NE
ER+	CCDC144NL-AS1	4,20284	8.57E-11	1.20	1.00
	LINC01871	1,29741	3.65E-37	22.20	13.00
	MIAT	1,18386	2.27E-33	14.10	6.70
	SNHG9	1,12129	1.71E-45	22.90	12.40
	MRPS30-DT	-2,51846	1.11E-05	0.20	1.20
TNBC	BHLHE40-AS1	2,14545	6.21E-21	5.00	12.00
	CYTOR	1,02092	7.89E-52	35.10	21.60
	LINC01229	8,41529	1.50E-14	1.90	0
	LINC01480	2,28935	3.16E-20	4.50	0.90
	LINC01871	2,09189	6.90E-70	26.60	12.80
	LINC01943	2,34382	2.10E-82	18.10	4.90
	LINC02099	3,78259	2.94E-27	4.20	0.40
	LINC02416	2,86265	1.90E-13	2.70	0.48
	LINC02446	2,34388	2.31E-73	20.10	7.20
	MIAT	1,07844	3.22E-17	14.00	7.90
	MIR155HG	2,31405	3.05E-59	19.20	7.70
	MIR3142HG	1,85442	4.43E-21	6.70	2.20
	MIR4435-2HG	1,79831	3.69E-103	29.00	11.00
	SCGB1B2P	8,86931	1.25E-54	6.20	0
	ATP2B1-AS1	-1,01995	3.61E-23	6.10	12.40
	LINC00402	-1,36025	1.35E-39	1.90	7.80
	LINC02256	-1,00721	2.14E-21	4.50	10.00
	MDS2	-1,36644	3.46E-15	1.40	4.30
	MZF1-AS1	-1,40058	7.56E-21	1.80	5.70
	OSER1-DT	-1,16168	3.46E-16	2.00	5.5
	SNHG7	-1,03243	2.91E-58	13.40	26.90

Note: The total number of cells evaluated was 4,925 in the ER+ E group and 5,645 in the ER+ NE group; and 5,589 in the TNBC E group and 4,137 in the TNBC NE group. E, potentially responsive to Pembrolizumab treatment due to lymphocyte expansion; NE, potentially non-responsive to Pembrolizumab treatment due to limited lymphocyte expansion. *NA*: No lncRNAs met our identification thresholds.

Supplementary Table S3. LncRNAs expressed by dendritic populations identified in scRNA-seq data from ER+ and TNBC patients treated with pembrolizumab in pre-treatment time point.

BRCA subtype	lncRNAs	Average log2 fold changes	p-value adj	Cell expression fraction (%)	
				E	NE
ER+	EBLN3P	2,07526	9.29E-03	36.60	13.60
TNBC	*NA*	*NA*	*NA*	*NA*	*NA*

Note: The total number of cells evaluated was 93 in the ER+ E group and 235 in the ER+ NE group; and 191 in the TNBC E group and 323 in the TNBC NE group. E, potentially responsive to

Pembrolizumab treatment due to lymphocyte expansion; NE, potentially non-responsive to Pembrolizumab treatment due to limited lymphocyte expansion. *NA*: No lncRNAs met our identification thresholds.

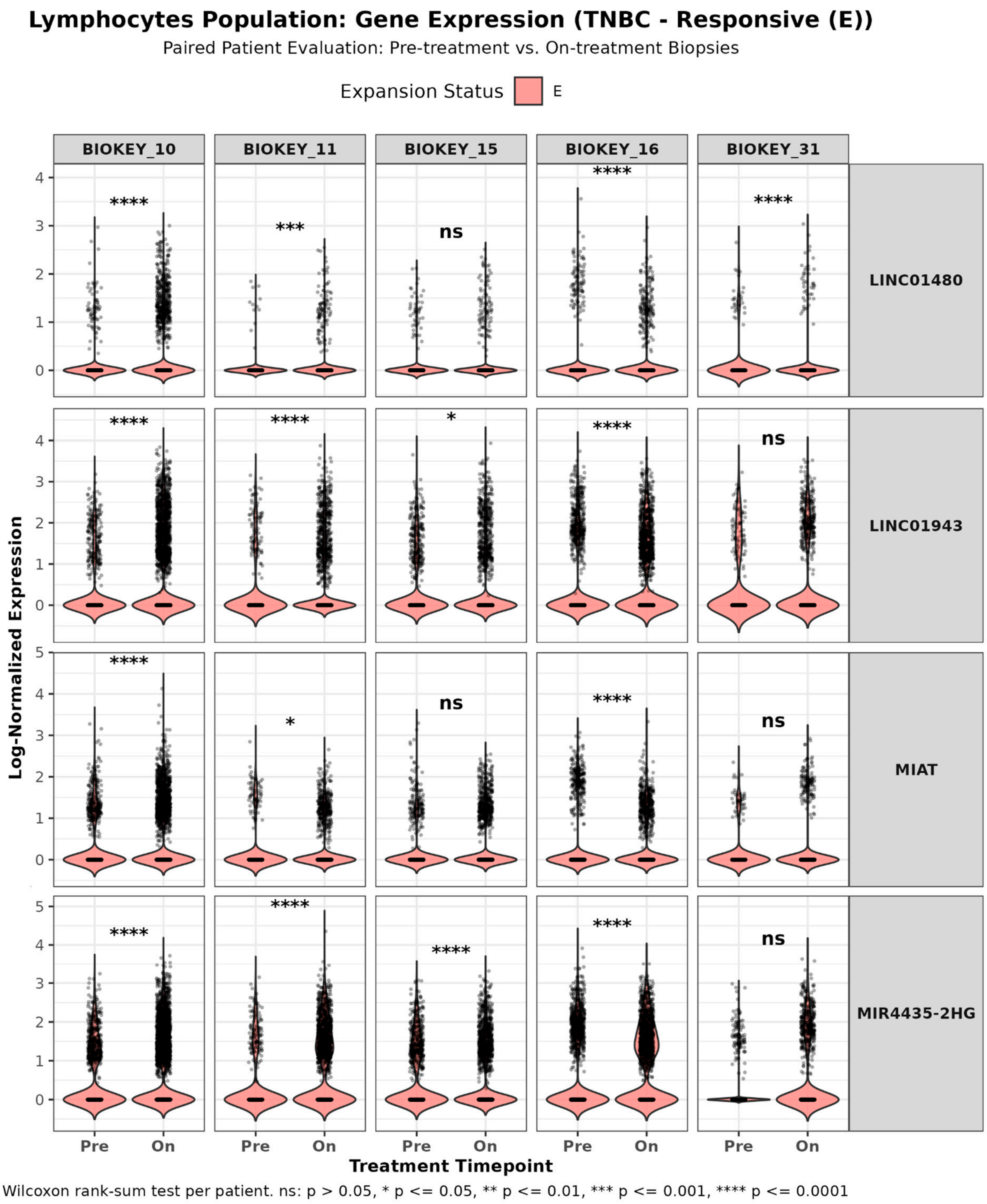

Supplementary Figure S9. Candidate lncRNA expression in lymphocyte populations from patients with triple-negative breast cancer (TNBC) at pre- and on-treatment time points with pembrolizumab. Violin plots showing the cellular-level expression of LINC01480, LINC01943, MIAT, and MIR4435-2HG by donor patient (Biokey). Patients exhibit clonal expansion of lymphocytes at the on-treatment time point (E group). Differences between groups were assessed using a two-sided

Wilcoxon rank-sum test: *p<0.05; **p<0.01; ***p<0.001; ****p<0.0001; ns, not significant. E, potentially responsive to Pembrolizumab treatment due to lymphocyte expansion.

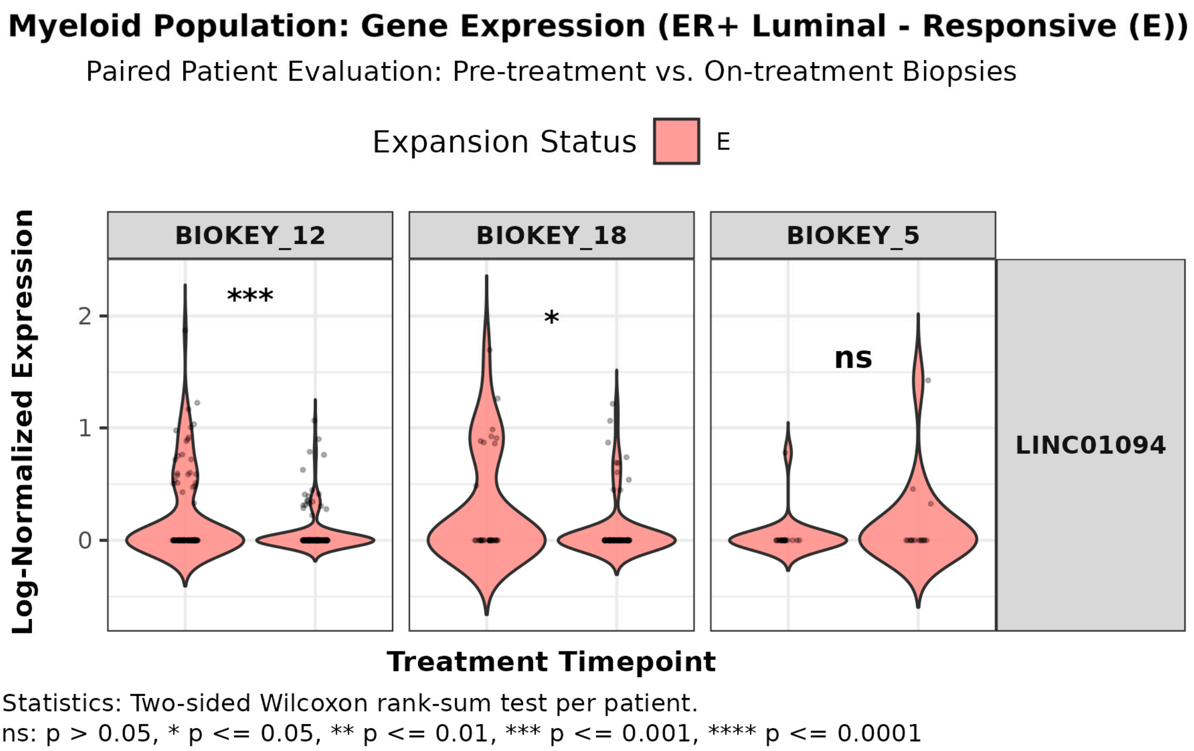

Supplementary Figure S10. Candidate lncRNA expression in lymphocyte populations from patients with luminal breast cancer (ER+) at pre- and on-treatment time points with pembrolizumab. Violin plots showing the cellular-level expression of LINC01094 by donor patient (Biokey). Patients exhibit clonal expansion of lymphocytes at the on-treatment time point (E group). Differences between groups were assessed using a two-sided Wilcoxon rank-sum test: *p<0.05; ***p<0.001; ns, not significant. E, potentially responsive to Pembrolizumab treatment due to lymphocyte expansion.
